# Usability and feasibility of an online intervention for older adults to support changes to routines and the home ('Light, activity and sleep in my daily life')

**DOI:** 10.1186/s12889-024-20309-y

**Published:** 2024-10-14

**Authors:** Kiran M. Gerhardsson, Mariam Hassan, Åsa B. Tornberg, Steven M. Schmidt

**Affiliations:** Department of Health Sciences, Lund University, Lund, Sweden

**Keywords:** Complex intervention, Behavioural change, Environmental proactivity, Wellbeing, Older adults, Usability, Feasibility

## Abstract

**Background:**

Indoor lighting, exposure to outdoor daylight, physical activity and sleep interact to influence functioning, mood and cicadian rhythm. Older adults (≥ 65 years), who often spend more time at home, are less physically active and experience more sleep problems, could benefit from strategies to support behavioural change and self-managed modifications in the home. The study’s primary objective was to assess the usability and feasibility of the ‘Light, activity and sleep in my daily life’ intervention, delivered as a web-based course.

**Methods:**

This 9-week intervention was delivered in a municipality in Sweden (55.70° N). Participants were eight healthy women (age 71–84), community-living in one-person households. We recruited through municipal staff and posters at senior citizen meeting points. Both qualitative and quantitative data were collected before and after the intervention. The outcome measures were intervention usability (ease of use, usefulness) and study feasibility (e.g., recruitment procedure, online engagement). Measures also included changes to routines and self-managed home adjustments to determine whether the participants applied what they had learnt.

**Results:**

All participants completed the intervention. Time logged in varied between 25 min and 3 h (*M* = 1 h 50 min) per week. Seven participants’ system usability scores were between 90 and 100 (‘Excellent’) out of 100. When interviewed, participants reported overall high satisfaction with what they had learnt. Six participants were particularly satisfied with the modules targeting light. Seven participants made changes to their lighting or darkness conditions, such as replaced bulbs with either 3-step dimming or higher colour temperature LEDs (samples were included in the intervention test kit). One suggestion to improve the online delivery was to enable participants to add text comments to the weekly evaluation form.

**Conclusions:**

The web-based intervention was feasible to deliver but time for recruitment should be extended and advertisement in the local newspaper should be considered. Participants’ computer proficiency and access to the internet at home will be critical in a future study with a larger sample. Only minor changes to the online content of the intervention are needed based on participants’ feedback. The intervention will be possible to evaluate in a future pilot study.

**Supplementary Information:**

The online version contains supplementary material available at 10.1186/s12889-024-20309-y.

## Background

Several behaviours have been found to positively influence health and wellbeing, such as engaging in regular physical activity [1], being exposed to light at the right time during the day [2, 3, 4] and maintaining regular sleep routines [5]. Research also indicates that indoor lighting, outdoor daylight exposure, physical activity and sleep quality interact to influence functioning, mood and circadian rhythm [2, 6, 7, 8, 9]. Light is the main external cue for synchronizing the internal circadian clock to the 24-h day. Inadequate light levels during the day can disrupt the circadian system, which can lead to a reduction in sleep quality. For example, the lack of daylight at higher latitudes may be involved in reported problems with seasonal changes in mood and sleep in winter [10, 11, 12, 13, 14]. However, the exact causes are still not known [2].

Older adults (age 65 and over) often spend more time at home after retirement [15], are less physically active [16] and experience more sleep problems [17, 18, 19]. Hence, they could benefit from strategies to support behavioural change and self-managed modifications related to lighting in the home. One study indicated that older adults have low lighting levels compared to lighting recommendations (approx. 200–500 lx) for visual tasks [20]. A recent pilot study including light measurements in the field (horizontally at the table and vertically at the eye level of the seated person) confirms that light levels are too low for both visual tasks and for stimulating a healthy circadian rhythm [21].

There are several developed health-promoting interventions directed at older adults targeting a single factor, such as physical inactivity [22] and sedentary behaviour [23, 24]. Few interventions target multiple behaviours simultaneously, such as inactivity, sedentary behaviour and sleep problems [25]. However, no intervention has, to our knowledge, targeted both behaviour and environmental proactivity, as well as multiple factors, such as physical activity outdoors and sleep routines. Environmental proactivity refers to persons whom themselves engage in modifying environments to live independently and healthy [26].

The ‘Light, activity and sleep in my daily life’ intervention was developed to promote wellbeing (e.g., better mood and sleep) and improve lighting and darkness conditions at home. The intervention content includes several components labelled according to the Behavior Change Technique Taxonomy [27], as shown in Table 1.

**Table 1:** Description of the intervention content (‘Light, activity and sleep in my daily life’)

Category*	Component	Examples from the intervention
Goals and planning	Goal setting (behaviour)	Self-selected goals, e.g., daily outdoor walks
	Problem solving	Participants are advised to identify barriers preventing them from changing routines, e.g., talking daily walks, and thinking of possible solutions, e.g., to walk with a friend
	Action planning	Participants are advised to plan beforehand when and where to act (implementation intention), e.g., taking an early morning walk directly after breakfast
Feedback and monitoring	Self-monitoring of behaviour	Self-recordings in sleep diaries and activity diaries
Social support	Social support	Face-to-face discussions with other participants at physical meetings to share experiences
Shaping knowledge	Instruction on how to perform a behaviour	On how to change sleep routines and increase the duration of daily walks
	Information about antecedents	On environmental and behavioural factors affecting sleep quality
Natural consequences	Information about health consequences	The effects of poor sleep quality on mood and individual functioning, environmental cues for setting the internal circadian clock
Regulation	Reduce negative emotions	Advice on accepting days of poor sleep or interrupted daily walk routines
Antecedents	Restructuring of the physical environment	Advice on basic lighting design to enable personalised changes to lighting and darkness conditions at home

^*^ Categories including behavioural change techniques with similar active ingredients, i.e., by the mechanism of change (not the mode of delivery) [27]

The intervention is delivered over nine weeks as a web-based course on a digital learning platform including three to four physical meetings. Course material is placed in nine modules covering electric lighting, daylight, physical activity outdoors and sleep. Each completed module ends with a brief online evaluation. Besides online material, the course includes a test kit containing light bulbs, a sleep mask, a checklist for the room inventory, a cap, a notebook, and a sleep diary. The purpose of the test kit is to encourage experimentation and provide printed copies to facilitate the completion of assignments.

A behavioural change intervention delivered as a web-based course has several potential immediate benefits, such as enabling participants to maintain good health by increasing control over their daily routines and home lighting conditions, and to develop their digital skills [28]. Potential intermediate benefits are fewer fall accidents at home because of better lighting conditions during the day and night, and readiness to use e-health applications [29, 30]. A potential long-term benefit is continued independent community-dwelling, participation in society and health-related quality of life. If the intended benefits are met and the implementation is feasible, such an intervention can benefit the older population's health behaviour. However, it is unknown whether an intervention delivered as a web-based course is an effective and acceptable mode of delivery for community-dwelling older adults. Such a delivery method includes several challenges, such as previous experience with computers or online education, internet use, and rapidly changing web-based interfaces [31]. Therefore, it is critical to create inclusive and easy-to-use online content.

The Technology Acceptance Model identifies two critical factors predicting technology adoption, for example, use of a computer, the internet or a software tool: ‘perceived usefulness’ and ‘perceived ease of use’ [32]. People will use an application if it is relevant to their needs. However, benefits might be outweighed by the effort to use the application. The model has been used in similar studies involving older adults [33, 34] and other studies examining older adults’ intent to use various technologies [35]. The usability of the ‘Light, activity and sleep in my daily life’ intervention was assessed in a previous lab study conducted in a full-scale model of an apartment including video monitoring [36]. Participants were invited to evaluate an earlier version of the intervention’s online content – experts in the first round and intended target users (community-dwelling adults aged 70 and over) in the second. The two-step usability evaluation proved valuable, and findings enabled refinement of the intervention’s online content and significantly reduced the number of identified usability issues. Changes to increase ease of use included, for example, clarifying the different types of text links, revising instructions and considering issues with online enrolment in the course. Participants in the second round also suggested that the intervention’s online content should be supplemented with physical meetings.

The primary objective of the current study was to evaluate the usability of the intervention and study feasibility in a real-world environment to examine how the intervention and study procedures will work in context. We specifically wanted to identify usability problems with the content of the intervention and its delivery on the digital platform, as well as determine whether the participants applied what they had learnt in terms of changes to routines and self-managed home adjustments. The focus is not on the intended intervention outcomes (activity and rest patterns, mood, behavioural skills, sleep, and lighting quality), but rather to determine if the outcomes were feasible to assess. The study is essential to further refine the intervention, recruitment and study procedures, as well as to estimate the participants’ commitment to complete the intervention. The study thereby enables a future larger study with an optimal design and evaluation of intervention outcomes.

## Methods

### Theoretical guidance

**Fig. 1:**
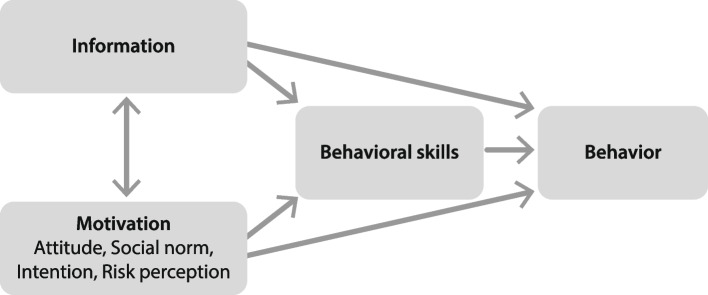
The original Information-Motivation-Behavioral Skills model (adapted from Fisher and Fisher [37])

The intervention strategy is based on the Information-Motivation-Behavioral Skills Model (see Fig. 1) that was developed for promoting health-related behaviour while considering social and psychological factors that influence such behaviours [37]. The model has been widely used for designing and evaluating behaviour change in several health-related domains [38, 39, 40]. The model identifies health-related information, motivation, and behavioural skills as being fundamental for people’s health-related behaviours. When people are well informed, motivated to act, and have the behavioural skills needed for effective action, they will likely initiate and maintain health-promoting behaviours and experience positive health outcomes.

In the ‘Light, activity and sleep in my daily life’ intervention, factual information is provided about, for example, light as the most potent external time cue for the internal body clock, characteristics of good indoor lighting, and the complex relationship between light, outdoor physical activity and sleep. To motivate participants, the intervention includes information about the individual benefits of maintaining routines and weekly encouragement from an interventionist when each module is completed. The intervention content also includes practical exercises and skills training to make learning experiences more interesting and enjoyable. Behavioural skills include practising fundamental lighting design, sleep restriction, and listing action plans to make goal-striving habitual behaviour [41, 42]. Behavioural changes involve physical activation, such as outdoor walking and changes to sleep routines.

### Intervention components

The ‘Light, activity and sleep in my daily life’ intervention:

- targets light-related behaviour, outdoor physical activity and sleep behaviour,
- considers multiple pathways (physical activity and cognitive goal setting and implementation) [41, 42],
- and is participant-centred by addressing self-identified needs, which can make the intervention more effective [43].

**Fig. 2:**
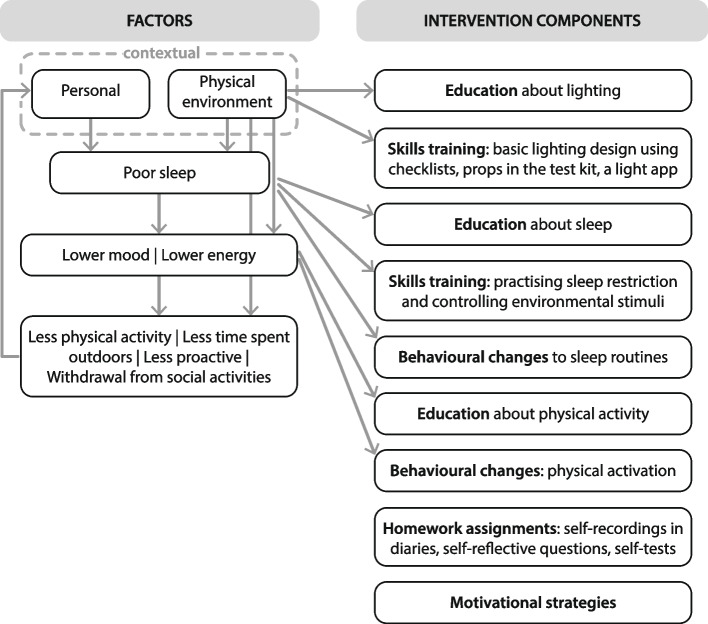
Conceptual framework – linking factors to intervention components

The general elements of the theoretical model in Fig. 1 are depicted in more detail in the conceptual framework in Fig. 2, linking the target of the intervention (contextual factors and consequences) to intervention components (information/education, behavioural skills/skills training and behavioural changes). Homework assignments involve applying what participants have learnt, which requires active participation, such as self-reflective questions (to support desired environmental changes and overcome possible obstacles to those changes), self-recordings in diaries, and self-tests. Another intervention component is external motivation through personal encouragement from an interventionist by sending personalised text messages on the phone after each completed course week and timely feedback on submitted weekly evaluations on the digital learning platform. Both personal and automated encouragement have been shown to support participants in proceeding with interventions in the context of internet cognitive behavioural therapy [44]. Still, additional motivational strategies are required to increase interest, sustain effort and persistence, and enhance self-regulation [45]. Interest and curiosity are sparked by providing relevant content, learning tasks that enable active participation and experimentation, and inviting self-selected goals and self-reflection. Sustained effort and persistence are supported by various resources (readings, text-to-speech, radio broadcasts, videos) and opportunities for peer interactions at physical meetings and mastery-oriented feedback (emphasising effort and practice rather than inherent abilities). Participants’ self-regulation, that is, effectively managing their engagement with the environment and emotional reactions, is supported by participant feedback and self-reflection.

### A mixed-methods approach

Both qualitative and quantitative data were collected before and after the intervention using a mixed-methods approach [46]. We collected information through questionnaires, semi-structured interviews, and field notes to identify usability problems in the intervention content and its delivery on the digital platform and to determine whether the participants applied what they had learnt. To evaluate study feasibility (recruitment and study procedure, retention rate, and online engagement), logged time was retrieved from the digital learning platform, and problems that we encountered during field work were recorded in field notes.

### Participants

Volunteers were recruited through posters, flyers, information meetings and personal social networks resulting in ten volunteers. Eight healthy women aged 71–84 (*Mdn* = 75.5) were selected in the order in which they gave written consent and completed a screening questionnaire. The number of participants was limited to eight. The limit was based on our experience from a previous usability study where six participants were sufficient for assessing the usability of the intervention in a full-scale model of an apartment [36]. Nielsen’s findings also support the idea that a low number of participants is sufficient to identify usability problems [47]. In addition, the available space for physical meetings was another limiting factor (the smallest room at the senior citizen meeting point could accommodate up to ten persons). The remaining volunteers were informed that they could participate in case anyone dropped out before the start of the intervention. Inclusion criteria were age 70 years or older, living in a one-person household in an apartment receiving little or no home care service, speaking Swedish, having access to a computer and smartphone, and being an internet user.

The invitation to participate in the study also stated that the course would be especially useful for those who experience at least two of the following problems: lacks suitable lighting or does not like their lighting environment, lacks routines for physical activity, usually has mild sleep problems, or experiences more fatigue, low mood or lack of energy during autumn/winter than during other seasons.

The screening questionnaire included questions about background characteristics, medication, general health, sleep routines during the past three months, diurnal preference and level of computer proficiency (CPQ-12, Computer Proficiency Questionnaire) [48]. Table 2 provides additional participant demographics and background information obtained from the screening questionnaire and interviews held during a home visit before the intervention. One of the participants lived in a house she owned, but she was not excluded from participation, ensuring a sufficient number of participants. One participant changed her mind about participation during the first week of data collection. She was replaced directly by a new participant who had given consent to participate in case anyone dropped out.

**Table 2:** Sample characteristics and background information

Category	Sample
Women^a^	8
Age^b^	75.5 (71, 84)
Level of education^a^	
… primary	1
… upper secondary	1
… vocational	1
… university	5
Swedish as …^a^	
… first language	6
… second language	2
Living in an^a^	
… apartment	7
… house	1
Housing tenure^a^	
… rental	2
… tenant-owned	4
… owner	1
Perceived general health score^b,c^	3 [3, 4]
Taking sleep medication^a^	
… no	5
… yes	3
Having severe sleep problems^a^	
… no	8
… yes	0
Self-reported diurnal preference^a^	
… extreme morning person	2
… more morning than evening	4
… neither	1
… more evening than morning	1
… extreme evening person	0
Computer proficiency^b,d^	22.3 [14, 29]

^a^*n*

^b^Median (range)

^c^In general, would you say that your health is: Poor = 1; Fair = 2; Good = 3; Very Good = 4; Excellent = 5

^d^Agreement with 12 statements about the ability to perform selected computer-related tasks (computer basics, printer, communication, internet, calendar, entertainment) with a computer, e.g. ‘I can use a computer keyboard to type’: Never tried = 1; Not at all = 2; Not very easily = 3; Somewhat easily = 4; Very easily = 5. A global score was computed with a minimum score of 6 and a maximum of 30. Two participants preferred to use a tablet. They were, therefore, asked to regard ‘computer’ as either a ‘computer or tablet’

### Data collection

#### Material

The primary outcome measures were intervention usability (ease of use and usefulness) and study feasibility (recruitment and study procedure, retention rate, and online engagement). Perceived usability of the web-based course was evaluated through paper questionnaires using the System Usability Scale (SUS) [49], shown in the Additional file 3. The purpose of obtaining the SUS scores was to supplement the participants’ verbal feedback on how easy it was to use the web-based course and their overall experience of the course. The SUS quantifies the usability of products and services, including software and websites, consisting of 10 statements on a 5-point Likert scale and one final question about the overall user-friendliness (adjective rating scale from 1 ‘Worst imaginable’ to 7 ‘Best imaginable’) [50]. Following published instructions on scoring of the 10-item SUS [51, 52], a total usability score is calculated ranging from 0 to 100 (SUS score). Higher SUS scores indicate greater usability. Participants’ general opinions of the intervention, its usefulness and perceived ease of use, and other concerns were provided by audio-recorded semi-structured interviews, partly inspired by the constructs of the Technology Acceptance Model [32] (see Additional file 1).

Changes to routines were identified through interviews. Self-managed changes in the home were identified through interviews and observations after the intervention (see Additional files 1 and 2). Observer-based environmental assessments (OBEA) of indoor features relating to lighting conditions during the first home visit were undertaken to identify potential environmental changes and serve as field notes.

Regarding study feasibility, page views and time logged on the course page (representing online engagement) were retrieved from the digital learning platform every Monday at 7 am. Recruitment and study procedure issues were identified in field notes. Intervention outcome measures (including activity and rest patterns, mood, behavioural skills, sleep quality and lighting quality) were collected to determine whether the selected data collection techniques were appropriate and manageable for the participants (see Additional file 4).

#### Setting and procedure

**Fig. 3:**
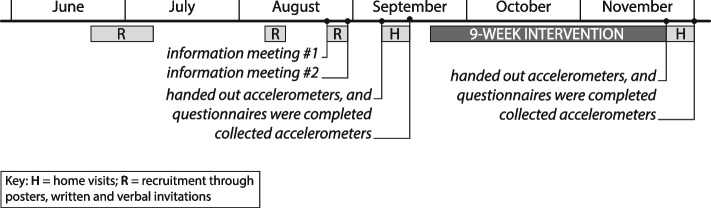
Study procedure

The 9-week intervention was delivered in a municipality in Sweden (55.70° N) during autumn 2022. The intervention started in late September because days get shorter after the autumn equinox at higher latitudes, and overcast days are more frequent in autumn, resulting in a significant drop in outdoor illuminance. The study procedure, from recruitment to data collection, is shown in Fig. 3.

Recruitment of intervention participants started after obtaining the ethics approval. Municipal staff (house hosts) distributed invitations to community-living residents in apartment blocks owned by the municipality. We used posters at the municipality-owned senior citizen meeting point in the city centre, the public library, a yoga studio and a thrift shop. We also recruited through verbal invitations at a public talk by a researcher at the senior citizen meeting point, community events and personal social networks, resulting in three persons attending the first information meeting. A second information meeting was held five days later because four persons could not attend the first one. Written consent and completed screening questionnaires were handed in by the persons who wanted to participate at the end of the meetings or collected by the researchers from one participant who could not attend the meeting.

Before the subsequent start meeting at the senior citizen meeting point, all participants had received an email with instructions for course enrolment and how to access the online learning platform. Eight participants, a researcher and a research assistant attended the meeting. The researcher started the meeting by asking the participants: 1) ‘Was there anything that worked well or less well in terms of the instructions?’, 2) ‘Is there anything you wonder about before we continue?’. The test kits were handed out, and participants received their accelerometer with an in-built light sensor (ActiGraph GT3X-BT, ActiGraph LLC, Pensacola, FL). They were instructed to wear it on the wrist of the non-dominant hand until the next meeting eight days later and to remove it only before showering, swimming or sauna bathing. They were advised not to cover the light sensor when they went outdoors. They also received a brief sleep diary for recording the time of going to sleep and awakening at home to facilitate the interpretation of future accelerometer data. Schedules were handed out to the participants, including physical course meetings and activities before, during and after the intervention. Each participant booked a convenient time for the home visit during the following seven days. At the end of the meeting, the participants completed four questionnaires (mood, behavioural skill, computer anxiety and sleep quality). The fifth questionnaire on lighting quality was to be completed in the evening after dusk at home by the participant seated at their favourite spot.

The subsequent home visit included audio-recorded interviews lasting 13–49 min and a series of open-ended questions related to their daily routines, home environment, physical activity, mood, and background characteristics (see Additional file 1). After the interview, a research assistant instructed the participant on how to use the digital learning platform. At the same time, the researcher recorded environmental features relevant to the lighting conditions using a prepared form (see Additional file 2).

After the home visits, another meeting was held at the senior citizen meeting point to collect the accelerometers, the completed brief sleep diary and provide additional instructions for communicating through the digital learning platform. The intervention started the following week (the third week of September) and ended after nine weeks in late November. Time logged in was recorded each week during the intervention.

The same data collection procedure as prior to the intervention was repeated during the last week of November, including an initial meeting (handing out accelerometers, scheduling home visits, completion of questionnaires), home visits (interviews and observations) and a subsequent meeting at the senior citizen meeting point (returning the accelerometers and the completed brief sleep diary). At the initial meeting after the intervention at the senior citizen meeting point, the participants were asked to evaluate the web-based course in a questionnaire (System Usability Scale) in addition to the four questionnaires. The interviews at home lasted 27–61 min. They included open-ended questions related to general opinions about the course content, usability concerns and changes to routines and in the home (see Additional file 1). Participants were not reimbursed for their participation in the study but could keep the test kit included in the intervention.

### Data analysis

#### Intervention usability and study feasibility

The 10-item scores on the System Usability Scale were analysed descriptively. Based on empirical evaluations of the SUS, a SUS score below 50 indicates usability difficulties, while scores in the 70 s and 80 s are considered promising [53]. A score of 68 was considered to be ‘OK’, between 68 and 80.3 was ‘Good’ and above 80.3 was ‘Excellent’.

Interviews from the home visits after the intervention were transcribed and analysed using a theory-driven approach based on the constructs of the Technology Acceptance Model [32]. Participants’ comments were sorted into the following pre-determined main categories: ‘Perceived ease of use’, ‘Perceived usefulness’ and ‘Course satisfaction’. Sub-categories were created inductively based on the participants’ feedback. Emergent findings were compared with field notes taken in connection with course enrolment and interactions with participants before, during and after the intervention.

The interviews also provided accounts of participants’ daily routines that were summarised into a graphic overview. Observer-based environmental assessments in the participants’ homes conducted during the home visits before the intervention were used to validate participants’ accounts of their environmental changes in the home at the home visit after the intervention.

The total time per week participants had spent on the course site on the digital platform was analysed descriptively. Weeks with no recorded activity were compared to field notes, such as if participants were away on travel.

## Results

### Intervention usability

Initial usability issues were identified during course enrolment. At the start meeting, before the home visits, all participants but one had managed to enrol in the course on the digital learning platform. When asked if they managed on their own after reading the e-mailed written instructions, it became clear that only three had successfully managed to enrol without assistance from adult children, grandchildren or the university administrator.

Seven participants’ SUS scores after the intervention were between 90 and 100 (‘Excellent’) out of 100 (see Additional file 6). Regarding the final question with an adjective rating scale in the SUS questionnaire, six participants rated the overall user-friendliness as 6 (‘Excellent’) out of 7, and two participants as 5 (‘Good’). Only one participant (P4) had a lower SUS score than 68, indicating poor usability. However, her response to the final question was 5 (Good). A tentative explanation for the lower SUS score of P4 was reflected by her agreement with the following statement:’I had to learn a lot of things before I could get going with this web-based course’ (one of the ten statements in the SUS questionnaire, see Additional file 3). In the interviews, one suggested alteration was to enable participants to add text comments to the weekly evaluation form to improve the online delivery.

Table 3 shows example quotes from the interviews after the intervention addressing the main categories of ‘Perceived ease of use’, ‘Perceived usefulness’ and ‘Course satisfaction’. In general, instructions on the digital course platform were not difficult to follow. Participants’ concerns were mainly related to the physical meetings in week 3 and 6 during the intervention. Some participants approved of while others questioned the choice of ‘meeting rules’ (raising one’s hand to speak instead of calling out and raising two hands to comment on a previous speaker) at the two physical meetings during the intervention.

**Table 3:** Example quotes from semi-structured interviews and examples from field notes. Numbers within parenthesis represent the prevalence of the sub-category across the participants, e.g., ‘[7]’ signifies that seven out of eight participants referred to the particular category

Category	Description	Example quotes (participant #)
*Perceived ease of use*		
Easy-to-follow online instructions but disagreement about meeting structure (7)	Written instructions on the digital learning platform were not difficult to follow. The choice of meeting technique was both appreciated and questioned (rising one hand before talking and two hands before addressing a previous comment). Some found time for the buzz group discussions too short, e.g., when discussing technical features of lamps or environmental changes in the home	About raising hands during the meetings: "My first reaction was that we are not children, but it was necessary to have some rules to follow." (P4) “Yes, I think it has worked well because it is needed, and you also see those who take up more space.” (P6)
Learning to navigate on the platform required some time (2)	One-on-one demonstration of the digital learning platform at the home visit was appreciated	“One thing that was very interesting to me was that when you explained, I understood and could do it myself and dared – even though I am so ignorant about computers.” (P4)
Previous computer use enabled easy navigation (5)	No problems navigating on the digital learning platform was related to previous experience with computers (but not to previous digital courses)	This participant did not experience any problems with finding one’s way on the digital learning platform: "No, I got where I wanted. But it was a bit cumbersome before I figured out how.” (P1)
*Perceived usefulness*		
Motivation to act (2)	A positive feeling of being pushed to deal with what needs to be changed regarding light in their home environment	“I knew I could improve the lighting and lamps, both practically and aesthetically, but I didn’t know how and didn’t take the initiative. So I am very happy I have almost finished how I wanted it.” (P2)
Increased awareness of lighting conditions and the effect of daylight on circadian rhythm, and knowledge about light sources (6)	The modules on light were more appreciated than others	“… we have increased our awareness and interest in how we want the light around us and what we enjoy. So I replaced that one [the lamp in the hall] because it was glary.” (P6)
Minor changes to routines relating to sleep rather than physical activity (7)	Participants were physically active before the intervention but made slight changes to some routines or felt motivated to continue current physical activities. Changes involved: morning walks were added or replaced walks later in the day, time-restricted eating, acceptance of two sleep periods during the night and quit taking sleeping pills	“… if you sometimes decide to relax through the day, you tell yourself to go outside now because it’s so important. And I can ride a bike even if my knee hurts, so you can bike anyway.” (P6) ”[The sleep diary] made me suddenly realise that at that time I should go to bed because then I fall asleep immediately—I never thought of that before.” (P7)
*Course satisfaction*		
Effortless course (3)	The course was not found to be demanding	
Slightly high workload (4)	A bit more time than expected was required for the course, e.g., listening to the radio broadcasts, or because some modules were a bit technical	”It has maybe been a little more to read than I had expected. I didn't put it in my schedule because I thought it would only take a few hours, but it has taken longer.” (P1)
Appreciation of in-person meetings (4)	Meeting physically twice during the intervention in weeks 3 and 6 was valued, and some were inspired by what other participants had done in their homes	”Yes, it was very good that we had such a walk-through. (P6)
High degree of freedom (2)	A largely self-directed course delivered online provides the freedom to choose when to read the material and to do the exercises	“I think the best thing is that it was at the right pace; you had plenty of time to think and decide for yourself how much time to spend […] I choose when I want to listen. It was quite relaxed.” (P2)
Variability in preferred learning media	Participants preferences for how to acquire information varied, such as listening to radio broadcasts or reading course content in print	One participant (P1) found a radio broadcast too long, whereas another enjoyed listening to all the broadcasts (P8). Four preferred reading paper printouts.

Based on the responses to the interview question, ‘How satisfied are you with what you have learnt?’, overall satisfaction was high. Two participants were satisfied, and six were very satisfied with their learning. Learning about light and the importance of keeping routines was mentioned by many, such as the ‘window for food intake’ and appropriate times for meals (P4) or taking an early morning walk (P1, P4, P7, P8). Meeting physically was valued (P4, P6). Two participants (P1, P5) specifically expressed that they were inspired by the changes other participants had made. An unexpected consequence of the intervention – increased self-awareness – was mentioned by one participant:

*“I think it has been very educational, and I have learnt a lot about myself. I think I noticed that in the others as well when we talked, that they learnt to think in a different way.” (P7).*

One participant (P8) had not made any changes in her home. Nevertheless, she expressed being ‘more satisfied than dissatisfied’ and particularly appreciated the links in the online course material to radio broadcasts. She realised her kitchen was too dark but did not feel motivated to make any changes. Neither was she motivated to change her sleep routines because she had accepted poorer sleep quality as a pensioner. She had, however, learnt to use a night feature on her smartphone and made attempts to take earlier walks in the mornings. Similar to many of the other participants, she already had several day-to-day activities scheduled. She had expected a much less demanding study in terms of time. In addition, her participation was affected by personal life events (planned medical surgery). Her stated reason for participating was that more volunteers were needed, and she wanted to contribute to the research.

Observations of participants’ self-managed home adjustments during the home visits after the intervention confirmed participants’ accounts. As displayed in Additional file 5, seven participants made changes to their lighting or darkness conditions, such as replacing bulbs with either 3-step dimming or higher colour temperature LEDs (samples were included in the intervention test kit), adjusting existing spotlights, installing luminaires, rearranging furniture or changing window treatments to allow more daylight to enter.

### Study feasibility

The recruitment procedure involved some challenges. Our target was to receive at least 20 volunteers to be able to select eight who met the inclusion criteria. The target was not met. Only ten persons volunteered, completed the screening questionnaire and gave written consent. All had received information about the study in different ways: a poster at the senior citizen meeting point (*n* = 1), at the public lecture at senior citizen meeting point (*n* = 1), through the invitation from the house hostess (*n* = 1), at a community event (*n* = 1), through personal networks (*n* = 1), or an invite from another participant (*n* = 5). Eight participants enrolled and completed the intervention.

Despite describing the inclusion criteria in posters and invitations, all volunteers did not meet the criteria, for example, having access to a computer and smartphone, and being an internet user. After the start meeting, it became clear that one participant did not have access to either a computer or smartphone (she had, however, gotten hold of a smartphone from her brother before the home visit). She had a tablet but did not have the PIN code to open it. She was advised to go to the supplier and ask them to open the tablet. At the home visit, we realised the tablet needed a complete software upgrade and that the internet was not accessible. To continue her participation and avoid paying for internet access, she agreed to sit at the senior citizen meeting point, where she did each weekly module using their wireless internet connection. (In rented municipality-owned apartments in Sweden, internet access must be paid for by the tenant.)

The Computer Proficiency Questionnaire handed out at the senior citizen meeting point included statements about, for example, computer use. The questionnaire proved valuable for indicating the participants’ level of computer proficiency. One participant, whose score was in the lower half (below 18), showed great difficulties using her tablet and smartphone during the home visits and the intervention (see the Additional file 6).

Flexibility is something to consider when delivering an intervention on a digital learning platform. Five participants preferred reading print on paper but two of them did not have access to a printer at home. The delivery procedure was adjusted so they could come to the senior citizen meeting point every Friday afternoon during the nine-week intervention to collect printed material for the next module (7 to 14 pages). Another deviation from the planned procedure was the weekly one-to-one support for one participant, lasting about 40 min. The participant was asked to go through the weekly module and think aloud when she submitted her responses to the weekly evaluation or the online exercises.

Some steps of the study procedure could be improved. For example, we handed out the test kit included in the intervention material prior to the home visits before the intervention, which led to four participants using the extended sleep diary and one participant using the light bulbs before the intervention had started. To avoid confusion, the test kit should be handed out at the meeting after the home visits.

Learnings from the data collection prior to the intervention delivery included that recording a detailed sleep diary with timestamps for all awakenings can be stressful for some (which was the reported reason by the participant who dropped out on the second day of data collection), and using a wristband with Velcro closure to attach the accelerometer can be annoying because it can damage clothes. The participants appreciated using one place for all meetings (the senior citizen meeting point).

Analyses from the wrist-worn accelerometers, which had in-built photopic light sensors, showed issues with the light readings. For example, the number of minutes exposed to 1000 lx and above was only 13 min for one of the participants after the intervention, indicating that the light sensor was likely covered by clothing. Regarding results from the questionnaires asking about mood (core affect), we learnt that life events, such as a fall accident in the family or a planned medical surgery, may negatively impact both dimensions of core affect (valence and activation). However, the subsequent interviews in the home provided an opportunity to follow up self-ratings on mood.

All eight participants completed the intervention and attended the physical meeting during the sixth intervention week. Seven attended the physical meeting during the third week (one was absent because of travel). Time logged in varied between 25 min and 3 h (*M* = 1 h 50 min) per week.

Descriptive results from the questionnaires are shown in Figures c–h in Additional file 6.

## Discussion

According to the Information-Motivation-Behavioral Skills model, both health-related information and motivation activate behavioural skills, which results in behavioural change and maintaining change, or activate behavioural change directly [37]. In their model, motivation includes, for example, attitudes, social norms or perceptions of risk. The reported findings from the evaluation of the ‘Light, activity and sleep in my daily life’ intervention reflect that motivation is also critical for approaching or avoiding learning activities. The participants’ motivation to engage in learning activities, or lack thereof, and their perceptions of how useful the course was for them are captured by ‘task value’, one of two critical components of the Expectation-Value model [54]. Both ‘expectation of success’ and ‘task value’ are predictors of performance and choices. ‘Task value’ is of particular interest in this context reflecting the perceived attractiveness of a task. ‘Task value’ consists of four components that can be linked to the accounts of the participants of this intervention: interest value (or task enjoyment), utility value (or usefulness to one’s goals, finding the intervention to be ‘important’), attainment value (importance to the self), and costs (psychological barriers or negative consequences of task engagement, such as time, effort or feelings of anxiety).

The four components of ‘task value’ capture the lack of motivation in one of the participants (P8). She had very limited interest in the learning activities (low interest value). She explained when she started that she volunteered because she was asked by another participant and friend who helped us recruit additional participants. She did not consider herself to match the target group but wanted to contribute to this type of research. She did not want to change her sleep routines because she had accepted poorer sleep quality as a pensioner, and she did not want to try any of the new types of LED bulbs included in the test kit (low utility value). Unlike the other participants, who unexpectedly wanted ‘course diplomas’, it did not seem to be important to her self-identity to do the learning activities (low attainment value). The participant stated that she did not have much time to engage in the learning activities as she was already engaged in many other organised activities (high costs). This single case illustrates that in behavioural change intervention research, it is more optimal to find volunteers who want to participate for the targeted reasons and are ready to change their current behaviour [55]. The interventionist can then support them to increase their motivation in several ways, as mentioned in the description of the intervention components in the background section. The findings show that participants can complete a complex intervention without being equally interested in all three topics (light, activity and sleep).

The fact that all but one had made some changes in their home environment regarding lighting suggests that the intervention was useful for the participants. High usability was also indicated by high SUS scores and overall high satisfaction with what the participants had learnt. The physical meetings appear to have positively influenced the participants' level of satisfaction. One study in a Swedish context found two dominant reasons for older adults to engage in educational activities: 1) staying active and 2) socialising [56]. Therefore, a hybrid and not an online-only intervention seems to be the right choice.

Participant recruitment must start earlier than late June to improve study feasibility and ensure a sufficient number of volunteers. Summer months are not ideal for recruitment in countries in which this period is a peak time for vacations and holiday travels even after retirement. Also, additional recruitment strategies should be considered to avoid poor recruitment, (see, for example, [57]).

The following should be added to the procedure to improve study feasibility and ensure participants meet the inclusion criteria: a short phone call after completion of the screening questionnaire to confirm that volunteers have access to an internet connection at home and know how to use it by asking a question about when they use the internet at home. A detailed answer could confirm they can access and use the internet at home. It would be too late to wait until the home visit and the walk-through of the digital learning platform to check internet access and computer proficiency. The purpose of a short screening interview is in this case to evaluate volunteers “to clarify their screening-survey responses and validate whether they are a good fit” [58]. Another option, in case of difficulty in hearing, is to include a question in the screening questionnaire (‘How often do you use the internet at home?’).

The questionnaires measuring the intervention outcomes were distributed before the home visits and the collection of accelerometer data. The reason was that responses to the questionnaires (e.g., on mood) could be followed up in the subsequent interview at the home visit. However, two questionnaires (sleep quality and mood) ask the participants to report their sleep and mood for the past few days. In a future study, accelerometry and self-reported sleep quality should ideally be assessed for the same period to enable comparison. Therefore, the sleep quality and mood questionnaires should be distributed a second time after collecting accelerometer data. Research has shown that people underreport their total sleep time compared to accelerometry [59]. Furthermore, the association between accelerometer-measured sleep and self-reported sleep quality is low [60]. Despite low associations, self-reported sleep quality is valuable. Studies suggest that the two measurement methods capture different dimensions of sleep [61, 62]. Studies also indicate that people’s subjective experience of their night sleep and reported sleep period predict higher positive affect the next day [63].

The light readings did not provide sufficient information about the participants’ duration of outdoor daylight exposure. When data were collected in late November, the in-built light sensor was probably covered by clothing, or outdoor illuminance was lower than anticipated (< 1000 lx). In a future study, participants will not be asked to uncover the accelerometer when they are outdoors. Rather, an activity diary could be an option for recording outdoor daylight exposure, but it will increase participant burden. An in-built light sensor can still be beneficial for analysing active periods during nighttime hours, such as for participants with a pattern of two periods of nighttime sleep, including an in-between wake period (for example, laying still reading in bed).

### Strengths

The study was characterised by an interdisciplinary approach combining architecture, psychology, physiotherapy and public health, which enabled a more complex intervention in terms of targeted factors and richer data. Deploying multiple techniques for collecting both quantitative (questionnaires) and qualitative data (interviews) made it possible to check reliability and interpret questionnaire results, for example, when participants showed negative changes in mood. Another strength of the study design is the combined use of self-reported questionnaire data and performance data (accelerometer-measured activity and rest patterns). Logged-in time on the digital learning platform enabled comparison with self-reported time in the weekly online course evaluations and field notes.

A strength regarding the sample was the diversity of participants’ backgrounds (e.g., education, first language) and previous experience with computers, which contributed to identifying more usability issues than would have been possible with a more homogenous sample. Sample size varies greatly in qualitative studies depending on the study aim [64]. The sample size in the current study was sufficient considering the study purpose and the theory-informed qualitative data analysis. We wanted to capture explicit, concrete issues in the qualitative data, not subtle or conceptual ones [65].

### Limitations

Although the sample size was small, it enabled adjustments to procedures to meet individual needs, such as providing one-to-one support at the senior citizen meeting point. However, attending to such needs will not be possible when the intervention is delivered in a larger study in the future. Therefore, the participants’ internet access at home and computer proficiency must be thoroughly checked during the screening before enrolment. Like any web-based intervention, the mode of delivery is a limitation in settings (districts or buildings) where internet access is either lacking or unreliable.

Based on interviews and the accelerometry data, the participants were physically active before the intervention started according to WHO guidelines [1]. A broader sample, including less physically active participants, is needed to determine whether the intervention can positively impact changes in activity and rest patterns. Repeated assessments at more than one point after the intervention will show whether changes are maintained.

Logged-in time on the digital learning platform may not accurately reflect the time spent reading/listening to the online course material and doing the exercises. Despite asking the participants to always log out, they might have stayed logged in when doing other non-course activities. Some of the participants commented that it was not always easy to estimate the time spent on each course module in the weekly online evaluations since they also read material on paper printouts.

The intervention was provided in one municipality. There may be other potential issues when conducting a context-dependent intervention study. Consequently, additional sites should be included in a future pilot study.

## Conclusion

The web-based intervention was feasible to deliver, but time for recruitment should be extended, and advertisement in the local newspaper could be considered to reach a wider group of potential volunteers. The feasibility of a future study with a larger sample will heavily depend on participants’ computer proficiency and internet access at home. Concerning the intervention’s online content, only minor changes are needed based on participants’ feedback. Given that the identified challenges are managed, the intervention will be possible to implement and evaluate in a future pilot study because of the relevant, easy-to-use content.

## Supplementary Information

**Supplementary Materials 1. Semi-structured interview guide**

**Supplementary Materials 2. Observer-based environmental assessment form**

**Supplementary Materials 3. Usability testing questionnaire**

**Supplementary Materials 4. Intervention outcome measures**

**Supplementary Materials 5. Examples of self-managed changes in the home**

**Supplementary Materials 6. Descriptive graphs**

**Supplementary Materials 7. Accelerometry results**

## Data Availability

The datasets analysed during the current study are available from the corresponding author on reasonable request.

## Abbreviations

CPQ-12: Computer Proficiency Questionnaire
LED: Light-emitting diode
M: Mean
Mdn: Median
MVPA: Moderate-to-vigorous physical activity
OBEA: Observer-based environmental assessments
PA: Physical activity
PILQ: Perceived indoor lighting quality
SB: Sedentary behavior
sSCAS: Short Swedish Core Affect Scales
SE: Sleep efficiency
SUS: System Usability Scale
TST: Total sleep time
WASO: Wake after sleep onset
