# Supplementary material for: Usability and feasibility of an online intervention for older adults to support changes to routines and the home ('Light, activity and sleep in my daily life')
Source: BMC Public Health. 2024 Oct 14;24:2808. doi: 10.1186/s12889-024-20309-y (PMC11475629; doi:10.1186/s12889-024-20309-y)
Supplement: Supplementary file 2 — Supplementary Materials 2. Observer-based environmental assessment form [file 12889_2024_20309_MOESM2_ESM.pdf]

Additional file 2: Observer-based environmental assessment

The purpose was to record design features influencing lighting conditions during the day and at night, and the possibilities for the participants to change light and darkness conditions. The assessment was done during the home visit before the intervention.

Participant (#)/date/time ……………………………………………………………...

Construction year/ceiling height ……...…………………………………………………

1. B L K	2. lamp W C	3. daytime EL CC	4. ext. shading device ES BA RO	5. curtain Lj/Da Sh/He	6. reflect. colours Low/ Inter/ High	7. wind. frame Lj/Da	8. room darke ning Ve, Ro, Bl	9. sill height (m)	10. window opening W x H (m^2)	10. X 0,8 (m^2)	12. floor W x D (m^2)	13. $Area_{gl}$/ $Area_{floor}$ (%)

0. Measure the room height
1. Room: bedroom (B), living room (L), kitchen (K)
2. Colour tone of the light source: warm (W), cool (C)
3. Daytime: electric lighting is turned on (EL), windows kept covered or shaded, e.g. closed blinds or curtains (CC)
4. Exterior shading device: external screen (ES), balcony above (BA) or roof overhang (RO)
5. Curtains: light (Li), dark (Da), sheer (Sh), heavy (He)
6. Reflective properties of floor and walls: low, intermediate or highly reflective
7. Colour of window frames: light (Li), dark (Da)
8. Room darkening: Venetian blind (Ve), roller blind (Ro), blackout curtains (Bl)
9. Window sill height, meters
10. Window opening: width (W) and height (H), meters
11. Multiply by 0,8 = glazing area
12. Floor area: measure width (W) and depth (D), meters
13. Glazing to floor ratio, $Area_{gl}$/$Area_{floor}$, indicating daylight conditions: small <12%, medium 12–18%, large >18%

In the room where the participant spends most of the day:

Mounting of ceiling pendant above the dinner table:

Is there a ceiling hook above the dinner table? Yes / No.

Position of the connection point: In the ceiling above the dinner table / in the ceiling close to the dinner table / on the upper wall close to the dinner table / missing.

Furnishing:

Is there plenty of space to move around in the room: Yes / Partly / No.

Objects in the window opening:

No objects / plants / luminaire / other objects

Is it okay to take a photo of the window opening?
