# Supplementary material for: Usability and feasibility of an online intervention for older adults to support changes to routines and the home ('Light, activity and sleep in my daily life')
Source: BMC Public Health. 2024 Oct 14;24:2808. doi: 10.1186/s12889-024-20309-y (PMC11475629; doi:10.1186/s12889-024-20309-y)
Supplement: Supplementary file 3 — Supplementary Materials 3. Usability testing questionnaire [file 12889_2024_20309_MOESM3_ESM.pdf]

Additional file 3: Usability testing questionnaire (System Usability Scale)

For each of the following statements, please mark one box that best describes your reactions to the online course.

1. I think that I would like to use the online course frequently.

   Strongly disagree ☐ ☐ ☐ ☐ ☐ Strongly agree

2. I found the online course unnecessarily complex.

   Strongly disagree ☐ ☐ ☐ ☐ ☐ Strongly agree

3. I thought the online course was easy to use.

   Strongly disagree ☐ ☐ ☐ ☐ ☐ Strongly agree

4. I think that I would need the support of a technical person to be able to use the online course.

   Strongly disagree ☐ ☐ ☐ ☐ ☐ Strongly agree

5. I found the various functions in the online course were well integrated.

   Strongly disagree ☐ ☐ ☐ ☐ ☐ Strongly agree

6. I thought there was too much inconsistency in the online course.

   Strongly disagree ☐ ☐ ☐ ☐ ☐ Strongly agree

7. I would imagine that most people would learn to use the online course very quickly.

   Strongly disagree ☐ ☐ ☐ ☐ ☐ Strongly agree

8. I found the online course very cumbersome (awkward) to use.

   Strongly disagree ☐ ☐ ☐ ☐ ☐ Strongly agree

9. I felt very confident using the online course.

   Strongly disagree ☐ ☐ ☐ ☐ ☐ Strongly agree

10. I needed to learn a lot of things before I could get going with the online course.

    Strongly disagree ☐ ☐ ☐ ☐ ☐ Strongly agree

11. Overall, I would rate the user friendliness of this product as:

    ☐ Worst imaginable
    ☐ Awful
    ☐ Poor
    ☐ Fair
    ☐ Good
    ☐ Excellent
    ☐ Best imaginable

Comments (optional):
