# Supplementary material for: Usability and feasibility of an online intervention for older adults to support changes to routines and the home ('Light, activity and sleep in my daily life')
Source: BMC Public Health. 2024 Oct 14;24:2808. doi: 10.1186/s12889-024-20309-y (PMC11475629; doi:10.1186/s12889-024-20309-y)
Supplement: Supplementary file 4 — Supplementary Materials 4. Intervention outcome measures [file 12889_2024_20309_MOESM4_ESM.pdf]

Additional file 4: Intervention outcome measures

Material

Table A1. Intervention outcome measures.

Category	Method of evaluation	Reference
Mood	Self-reported: Adapted version of Short Swedish Core Affect Scales (sSCAS)	(1)
Behavioural skill	Self-reported: A New General Self-efficacy Scale	(2,3)
Computer anxiety	Self-reported: Short Computer Anxiety Scale	(4)
Sleep quality	Self-reported: PROMIS 4-item Sleep Disturbance instrument	PROMIS Item Bank v1.0 (5)
Lighting quality	Self-reported:<br>Perceived indoor lighting quality (PILQ)	Derived from outdoor lighting scales (6)
Sleep (activity and rest patterns)	Performance-based measure using a wrist-worn accelerometer with an in-built photopic light sensor (ActiGraph wGT3X-BT). The light sensor can facilitate detailed sleep analysis.	
Sleep times	Self-reported: bedtime (turning lights out to go to sleep) and final awakening time using a brief sleep diary to be used as sleep period start and end times in the sleep analysis.	
Physical activity and sedentary time, steps and time outdoors (i.e., time exposed to >1000 lux)	Performance-based measure using a wrist-worn accelerometer with an in-built photopic light sensor (ActiGraph wGT3X-BT). An uncovered light sensor can help determine duration of outdoor daylight exposure.	
Changes to routines and learnings	Semi-structured interviews	Inspired by Davis (7) and Nie et al. (8)
Changes in the home environment	Observer-based Environmental Assessments (OBEA) + semi-structured interviews	

Table A2. Questions and items in questionnaires measuring intervention outcomes.

Category	Question or statement	Response format
Mood	How have you mostly felt for the past days? (three adjective pairs measuring the dimension 'valence', i.e. good or bad, and three measuring 'activation')	1- to 9-point scale:<br>'Very sad' to 'Very glad'<br>'Very dull' to 'Very peppy'<br>'Very depressed' to 'Very joyful'<br>'Very passive' to 'Very active'<br>'Very displeased' to 'Very pleased'<br>'Very sleepy' to 'Very awake'
Behavioural skill	Eight statements on: goal achievement, task accomplishment, obtaining important outcomes, succeeding at most any endeavour, overcoming challenges, effective performance, doing tasks well, performing well even when things are tough	Strongly disagree = 1; Disagree = 2; Neither agree nor disagree =3; Agree = 4; Strongly agree = 5
Computer anxiety	Eight statements, such as 'The harder I work at learning computers the more confused I get'; 'I have sometimes thought "Computers don't like	1 = Strongly disagree; 2 = Disagree; 3 = Mildly disagree; 4 = Mildly agree; 5 = Agree; 6 = Strongly agree

	me"; 'I can usually manage to solve computer problems by myself' (reversed scoring)	
Sleep quality	In the past seven days, my sleep quality was …	5 = Very poor; 4 = Poor; 3 = Fair; 2 = Good; 1 = Very good
	In the past seven days:<br>… my sleep was refreshing<br>… I had problem with my sleep (reversed scoring)<br>… I had difficulty falling asleep (reversed scoring)	5 = Not at all; 4 = A little bit; 3 = Somewhat; 2 = Quite a bit; 1 = Very much
Lighting quality	Five bipolar scales measuring the dimension 'Strength' and five measuring the dimension 'Hedonic tone': How do you perceive the light in this room?	1- to 7-point scale:<br>'Dark' to 'Light'; 'Weak' to 'Strong'; 'Unfocused' to 'Focused'; 'Drab' to 'Clear'; 'Subdued' to 'Brilliant'; 'Cool' to 'Warm'; 'Hard' to 'Soft'; 'Unnatural' to 'Natural'; 'Sharp' to 'Mild'; 'Glaring' to 'Shaded'
	How well can you see in this light?	1- to 7-point scale:<br>'Very poorly' to 'Very good';

Participants' scores from the adapted version of the short Swedish Core Affect Scales were converted to a 'valence' score (bad/good) and an 'activation' score, resulting in a total score for each dimension ranging from 3 to 27. Participants' scores on eight items in the New General Self-Efficacy Scale were summed and divided by eight, resulting in a global score ranging from 1 to 5. Participants' scores on six items in the Short Computer Anxiety Scale were summed, resulting in a global score ranging from 6 to 36. Participants' scores from the PROMIS 4-item Sleep Disturbance instrument were summed, resulting in a raw score ranging from 4 to 20. Using a conversion table (68), the raw score was then translated to a total raw score (T-score) ranging from 32 to 73.3. Participants' scores on five items measuring the dimension 'strength' in the Perceived Indoor Lighting Quality (PILQ) questionnaire were summed and divided by five, resulting in a total score, ranging from 1 to 7. The scores on five PILQ items measuring the dimension 'hedonic tone' were summed and divided by five, resulting in a total score, ranging from 1 to 7.

Data analysis of intervention outcome measures

Data from the questionnaires were analysed descriptively in Stata version 17.0 by comparing individual participants' scores from baseline to after the intervention. Accelerometer-measured activity and rest patterns were analysed using the software ActiLife v613.4 to document changes from baseline by subtracting the post-intervention measurement from the baseline measurement on sedentary behaviour, number of steps and physical activity (light physical activity and moderate-to-

vigorous physical activity (MVPA)). The software ActiLife v613.4 was used to analyse changes in sleep behaviour from baseline by subtracting the post-intervention measurement from the baseline measurement (total sleep time (TST), sleep efficiency (SE), wake after sleep onset (WASO), number of awakenings and minutes exposed to 1000 lux and above). Accelerometer data was missing or disregarded for two participants (P2, P8) because one joined later in the week when accelerometer data were collected before the intervention and the other had a planned medical surgery (unrelated to the intervention) affecting her behavioural patterns during the data collection after the intervention.

References

1. Västfjäll D, Gärling T. Validation of a Swedish short self-report measure of core affect. Scand J Psychol. 2007;48:233–238.

2. Chen G, Gully SM, Eden D. Validation of a new general self-efficacy scale. Organ Res Methods. 2001;4:62–83.

3. Bandura A. Self-efficacy: The exercise of control. New York: W. H. Freeman; 1997.

4. Lester D, Yang B, James S. A short computer anxiety scale. Percept Mot Skills. 2005;100:964–968.

5. PROMIS Sleep Disturbance Scoring manual. https://forms.loinc.org/75258-4 (2023). Accessed 23 Dec 2022.

6. Johansson M, Pedersen E, Maleetipwan-Mattsson P, Kuhn L, Laike T. Perceived outdoor lighting quality (POLQ): A lighting assessment tool. J Environ Psychol. 2014;39:14–21.

7. Davis FD. Perceived usefulness, perceived ease of use, and user acceptance of technology. MIS Quart. 1989;13:319–340.

8. Nie Q, Nguyen LT, Myers D, Gibson A, Kerssens C, Mudar RA, et al. Design guidance for video chat system to support social engagement for older adults with and without mild cognitive impairment. Gerontechnology. 2020;20:1–15.
