# Supplementary material for: Usability and feasibility of an online intervention for older adults to support changes to routines and the home ('Light, activity and sleep in my daily life')
Source: BMC Public Health. 2024 Oct 14;24:2808. doi: 10.1186/s12889-024-20309-y (PMC11475629; doi:10.1186/s12889-024-20309-y)
Supplement: Supplementary file 5 — Supplementary Materials 5. Examples of self-managed changes in the home [file 12889_2024_20309_MOESM5_ESM.pdf]

Additional file 5: Examples of self-managed changes in the home

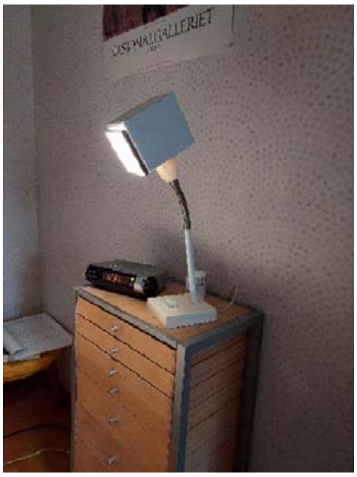

Image #1

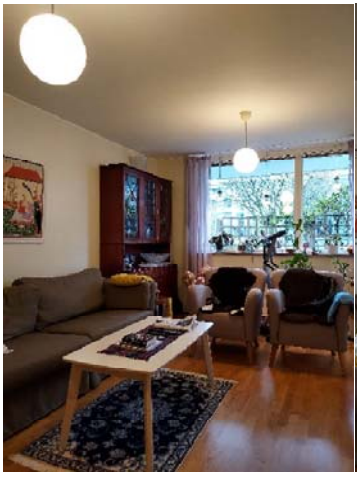

Image #2a

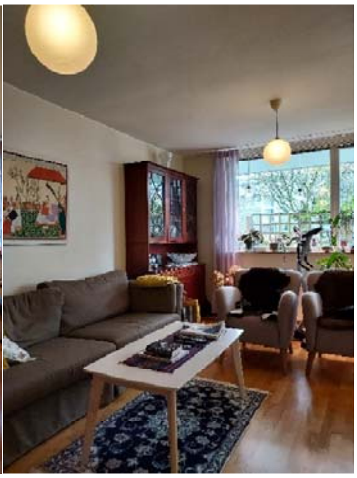

Image #2b

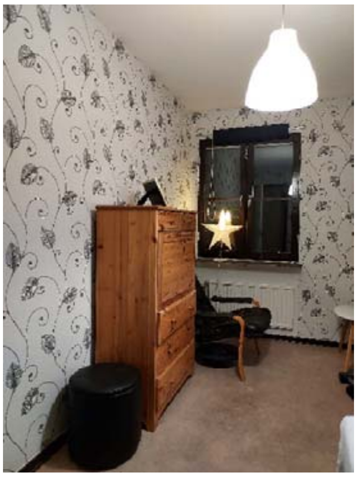

Image #3

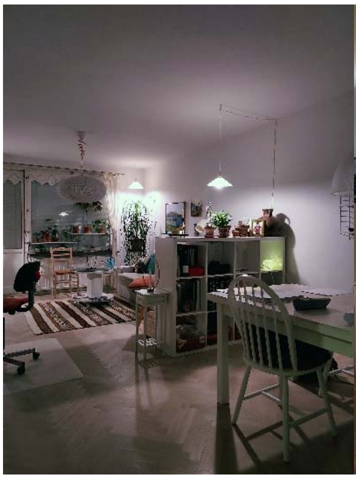

Image #4a

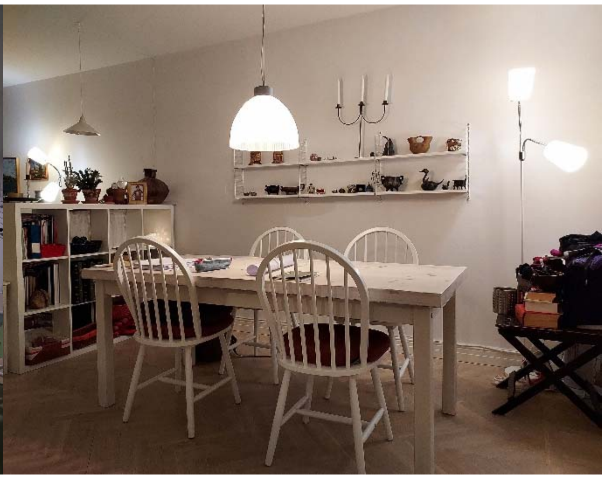

Image #4b

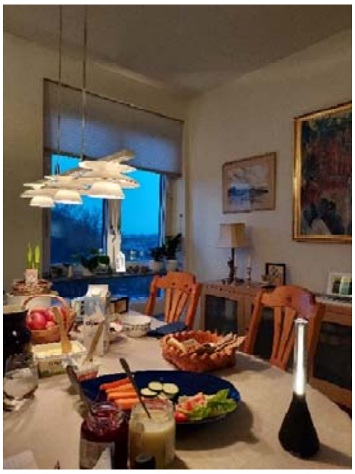

Image #5

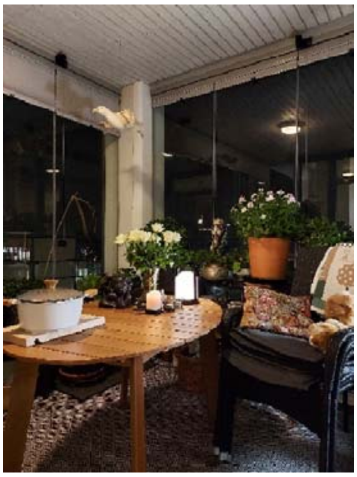

Image #6

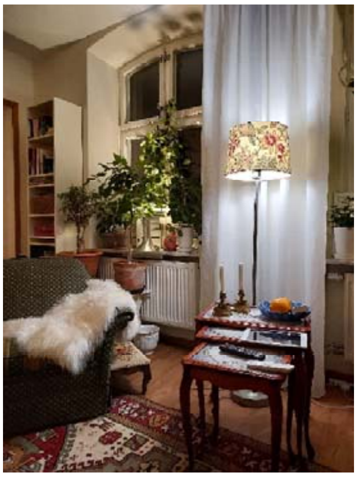

Image #7

Self-managed changes included, for example, replacement of the bulb to an LED bulb with a higher colour temperature (#1; #3; #7), replaced bulbs with 3-step dimming LED bulbs (#2a,b; #4b), replaced a rug with a light-coloured one (#3a), removed things on the window sill to allow more daylight (#5), hired an electrician to adjust a dimmer switch on the wall for LED-bulbs, replaced a dining table cloth with a light-coloured one (#5), bought a battery-powered portable luminaire (#5; #6), and rearranged furniture to allow more daylight to enter (#7). Photos were taken in the homes of seven participants by the first author during the home visit after the intervention.
