# Supplementary material for: Usability and feasibility of an online intervention for older adults to support changes to routines and the home ('Light, activity and sleep in my daily life')
Source: BMC Public Health. 2024 Oct 14;24:2808. doi: 10.1186/s12889-024-20309-y (PMC11475629; doi:10.1186/s12889-024-20309-y)
Supplement: Supplementary file 6 — Supplementary Materials 6. Descriptive graphs [file 12889_2024_20309_MOESM6_ESM.pdf]

Additional file 6: Descriptive graphs

Self-rated measures and comments.

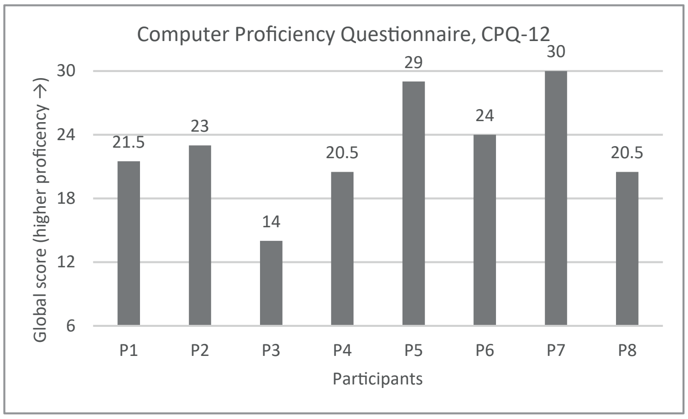

Figure a.

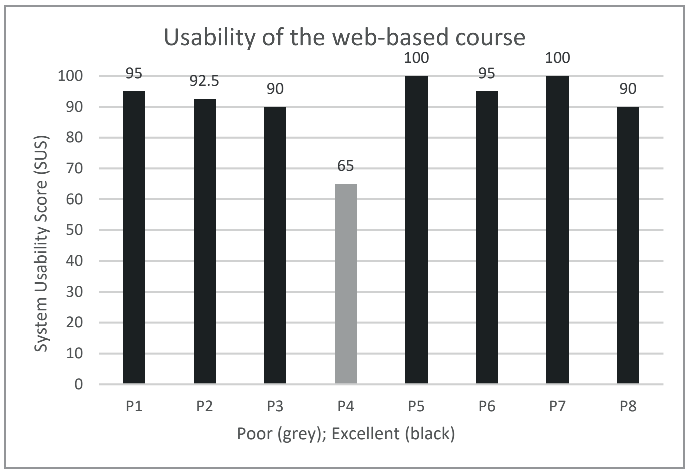

Figure b.

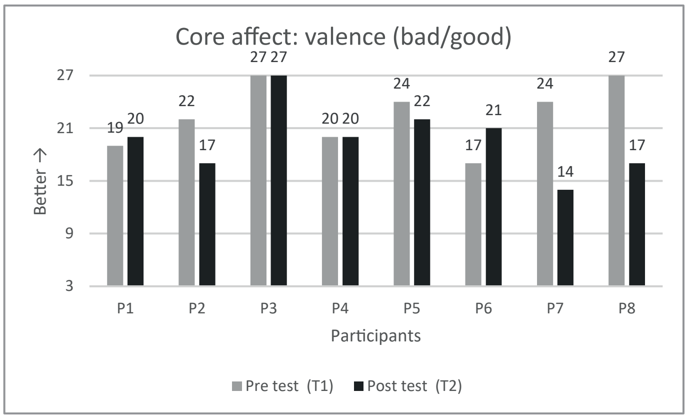

Figure c. As reported by the participants, life events had affected P7 (fall accident in the family) and P8 (surgery) during the intervention

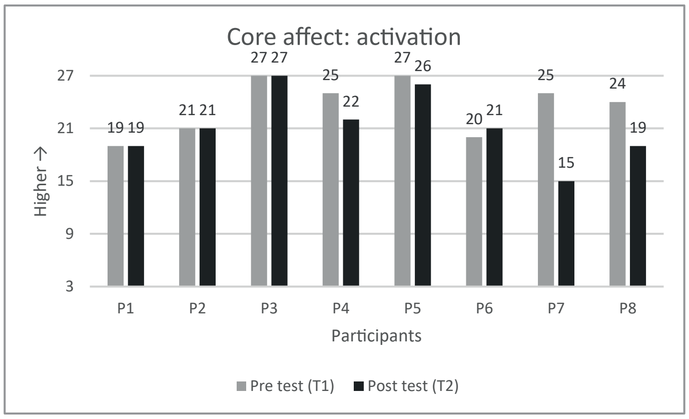

Figure d. As reported by the participants, life events had affected P7 (fall accident in the family) and P8 (surgery) during the intervention

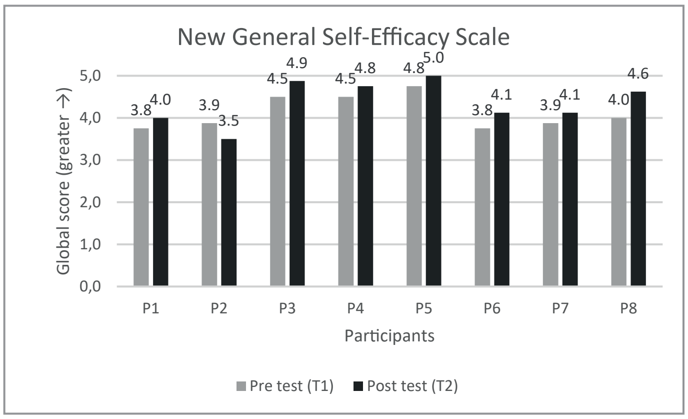

Figure e. Self-efficacy improved slightly after the intervention for seven participants

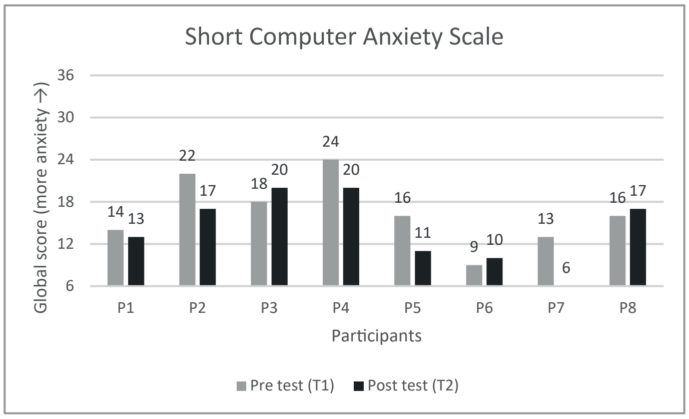

Figure f. There was an apparent decrease in computer anxiety in half of the participants after the intervention

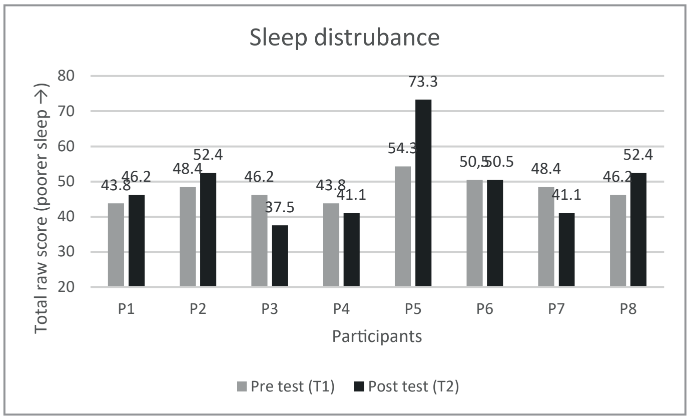

Figure g. There was a large change in sleep disturbance for P5, who reported day-to-day stress trying to get hold of artisans early in the mornings

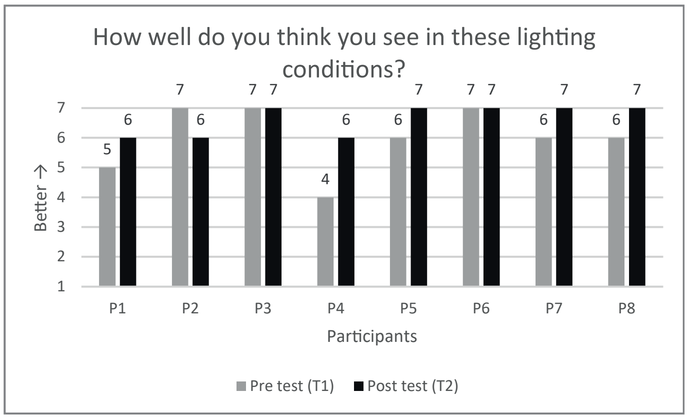

Figure h. Five participants reported seeing better at their favourite spot after the intervention, although changes were minor
