# Supplementary material for: Usability and feasibility of an online intervention for older adults to support changes to routines and the home ('Light, activity and sleep in my daily life')
Source: BMC Public Health. 2024 Oct 14;24:2808. doi: 10.1186/s12889-024-20309-y (PMC11475629; doi:10.1186/s12889-024-20309-y)
Supplement: Supplementary file 7 — Supplementary Materials 7. Accelerometry results [file 12889_2024_20309_MOESM7_ESM.pdf]

Additional file 7: Accelerometry results

Table. Change in behaviour from baseline (T1) to after the intervention (T2) shown as the number of participants increasing or decreasing sedentary behaviour, physical activity and sleep behaviour over the measurement period (7 days). Measurements from six participants were analysed.

Change in behaviour from T1 (baseline) to T2 (after the intervention)		
	Direction of change (observed range of change scores)*	T2<br>Number of participants
**Sedentary behaviour**, minutes/week	Increase<br>(500, 645)	2
	No change<br>(0)	0
	Decrease<br>(74, 297)	4
**Physical activity**		
Light physical activity, minutes/week	Increase<br>(257)	1
	No change<br>(0)	0
	Decrease<br>(241, 1030)	5
Moderate-to-vigorous physical activity, minutes/week	Increase<br>(77, 705)	4
	No change<br>(0)	0
	Decrease<br>(59, 384)	2
Number of steps/week	Increase<br>(275, 24665)	4
	No change<br>(0)	0
	Decrease<br>(3294, 11043)	2
**Sleep during the night**		
Number of sleep periods	Increase<br>(3)	1
	No change<br>(0)	2
	Decrease<br>(1, 2)	3
Sleep efficiency, %	Increase<br>(1.7, 2.0)	3
	No change<br>(0)	0
	Decrease<br>(0.5, 1.3)	3
Total sleep time (TST), minutes	Increase<br>(11, 89)	5
	No change<br>(0)	0
	Decrease<br>(31)	1

Wake after sleep onset (WASO), minutes	Increase<br>(3, 7)	3
	No change<br>(0)	0
	Decrease<br>(2, 8)	3
Number of awakenings	Increase<br>(3)	2
	No change<br>(0)	3
	Decrease<br>(2)	1

* All difference scores other than zero were treated as increases or decreases. Ranges represent minimum and maximum positive and negative changes in intervention outcome measures.
